# Identification of Plant Soot as Novel Safe Feed Additive: Evaluation of 90-Day Oral Toxicity and Prenatal Developmental Toxicity in Rats

**DOI:** 10.3389/fvets.2020.610627

**Published:** 2020-12-07

**Authors:** Xingyao Pei, Xilong Xiao, Jianling Liao, Linfu Ke, Daowen Li, Shusheng Tang

**Affiliations:** ^1^Department of Pharmacology and Toxicology, College of Veterinary Medicine, Agricultural University, Beijing, China; ^2^Fujian Shunchang Tanwawa Biological Technology Co., LTD, Shunchang, China; ^3^Fujian Plant Soot Biotechnology Co., LTD, Shunchang, China; ^4^Tianjin Key Laboratory of Agricultural Animal Breeding and Healthy Husbandry, College of Animal Science and Veterinary Medicine, Tianjin Agricultural University, Tianjin, China

**Keywords:** plant soot, subchronic toxicity, teratogenic, reproductive, embryo development

## Abstract

Plant soot, as a novel feed additive, could not only improve digestive function but also adsorb mycotoxins and inhibit bacterial infections. The subchronic toxicity and prenatal developmental effects of plant soot were studied for the first time. Our results indicated that there was no subchronic toxicity in the range of 2,000–50,000 mg/kg plant soot added in the feed, and there was no significant difference in reproductive function, embryo development, and teratogenicity between the pregnant rats exposed to 312.5, 1,250, and 5,000 mg/kg plant soot and the control group. The maximum no-observed effect level (NOEL) of supplemental dosage in feed could be set to 50,000 mg/kg, and the maximum intragastric NOEL could be set to 5,000 mg/kg, which preliminarily provided guidance on daily additive amount or clinical protocols for plant soot, as well as promoting the development and application of this harmless antibiotic substitutes.

## Introduction

**Figure 1:**
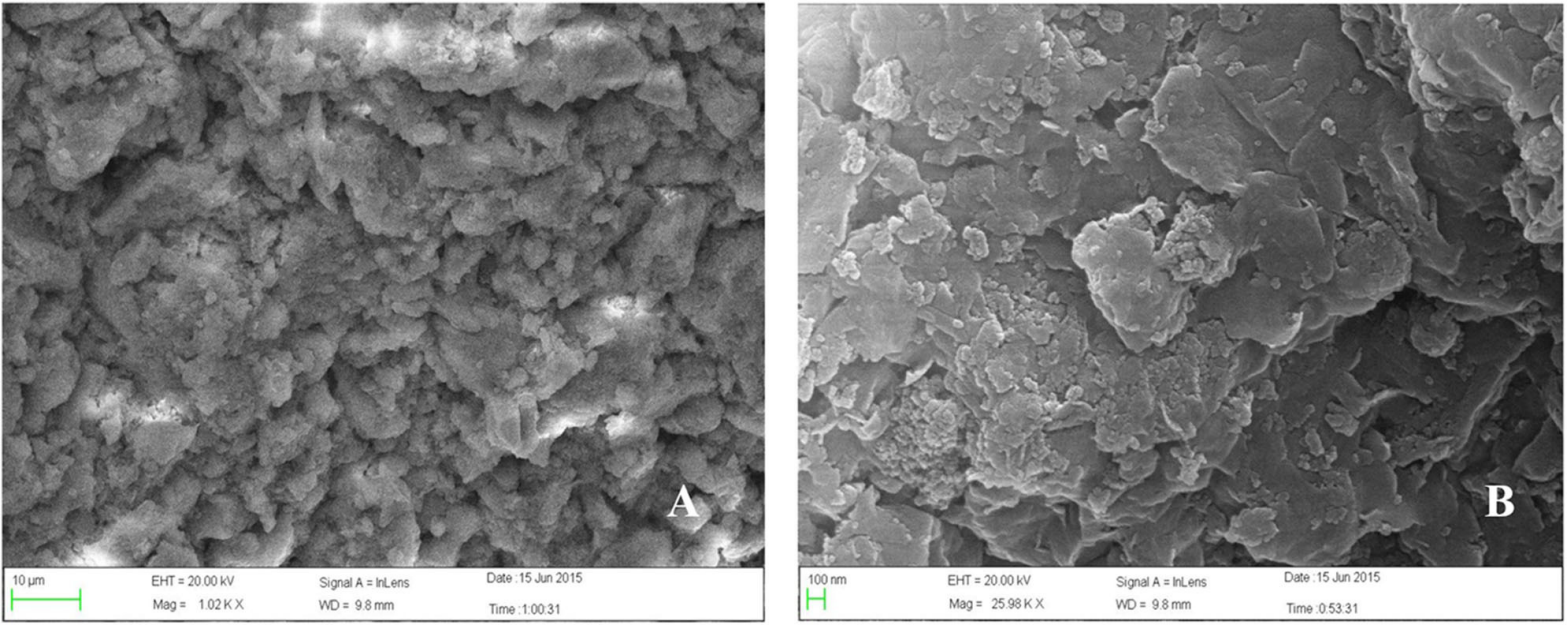
Scanning electron microscope (SEM) of plant soot. **(A)** The scale length is 10 μm. Magnification × 1,000. **(B)** The scale length is 100 nm. Magnification × 26,000.

In recent years, the prohibition and restriction of using veterinary antibiotics in livestock diets has become an important topic concerning human health. Most of the veterinary antibiotics have been forbidden as growth promoters in many countries due to the development of drug-resistant bacteria and the threat posed by drug residues, thus new antibiotic substitution strategies and alternative feed additives such as microecological preparations and herbal medicine is gradually becoming the mainstream trend (1, 2). One of the natural herbal medicines, plant soot, has been certified to adsorb intestinal mycotoxin, inhibit bacterial infection, and promote animal digestion, whose first industrial product has just been approved for use in feed additives by China in January 2020, without other details published concerning its toxicity analysis (3). The plant soot has been commonly known as “baicao cream,” which was the black powder produced by carbonization and activation products from bits of the Chinese fir, pine, or bamboo in raw wood processing. The source, production process, main ingredients, and main information of plant soot are shown in Table 1, and the microstructure of plant soot is observed in Figure 1.

**Table 1:** The source, production process, main ingredients, and main information of plant soot.

**Elements**	**Product information**
Generic name	Plant soot/plant carbon/Bai Cao Shuang
Raw materials	Sawdust of fir, pine, or bamboo left after wood processing
Processing methods	Carbonization and activation
Appearance and traits	Black powder, odorless, tasteless
Granularity (%)	Sieving rate (125 μm) ≥98.0
	Sieving rate (90 μm) ≥85.0
Dry decrement	≤12.0
Carbon content in dry basis (%)	≥90.0
Ash content (%)	≤8.0
Adsorption rate for 500 ng/ml zearalenone (%)	≥95.0 [treatment dose: 0.1% (w/v)]
Total arsenic (As)/(mg/kg)	≤4.0
Plumbum (Pb)/(mg/kg)	≤10
Mercury (Hg)/(mg/kg)	≤0.1
Cadmium (Cd)/(mg/kg)	≤1.0
Certificate authority	New Product Certificate for Feed and Feed Additives issued by Ministry of Agriculture and Rural Affairs of China (3)
Using realm	Approved to be produced, operated, and used in China

As an easily accessible and inexpensive herbal medicine, plant soot has been widely used in the medical field since ancient times. For example, early studies have shown that plant soot could relieve various pathological symptoms: (i) the bleeding was stopped and thus the symptoms such as hemoptysis, vomiting, hematemesis, and collapse were relieved; (ii) the diarrhea was reduced and the gastrointestinal digestion was improved, accompanied by increased increment in weight; and (iii) the symptoms including sore throat, mouth pain, and tongue headache could be reversed (4). Most importantly, recent research has suggested that oral plant soot effectively adsorbed intestinal toxins, especially mycotoxins, and inhibited bacterial infection, while external use of plant soot inhibited local bacterial infection and accelerated wound healing (5, 6). Furthermore, plant soot has been widely applied to the treatment of human and animal diseases such as enteritis, diarrhea, and acute tonsillitis (6). It has been demonstrated that animal diseases such as bovine nosebleed, bovine urine blood, bovine gastroenteritis, bovine oral ulcer, sheep scabies, rabbit mastitis, and piglet paratyphoid could be rescued and cured by plant soot (6). Between 2010 and 2012, the sterilization effect of plant soot was investigated after castration of healthy pigs from 2 months to 8 years old, then the cure rate increased from 92.4 to 97.3% compared with common anti-inflammatory drugs and disinfectants (7). In addition, the feed supplemented with 1–5% plant soot has been found to significantly increase the activity of pepsin, intestinal amylase, liver amylase, and non-specific immune enzymes in loach, with the significant rising of weight gain rate and growth rate. The most notable, loach enteritis and bacterial disease were effectively prevented by using plant soot, which was the first evidence for the antibacterial and growth-promoting effects of plant soot in aquaculture (8). Hence, plant soot has been expected to be widely used in animal husbandry and veterinary clinic, as well as becoming one of the ideal substitutes after the restriction policy on veterinary antibiotics.

At present, plant soot has become the only natural feed additive product that promotes the combination of wood waste carbonization and adsorbent. This unique combination has been proved to effectively promote animal growth and treat animal diseases. This natural feed additive has taken full advantage of the outcome that plants were artificially carbonized, converting urban waste timber into animal food supplement and medical energy. Then the ecological cycle was completed when they return to nature with animal excrement. Although most of the carbon derived from combustion waste has been demonstrated to pose immediate danger to human and animal health through inhalation (9, 10), it is reported that artificially some treated combustion or carbonized products had unique beneficial effect of adsorbing harmful substances from their surroundings (11). It needs to be clear that once the safety evaluation result of oral administration of plant soot is positive, the hazards of combustion and carbonization to human and animal health will change from absolute to relative. Up to now, there is extremely limited research information about plant soot, and the only existing analyses were mainly aimed at the medical value of plant soot, without the evaluation and analysis of toxicity. In our study, the safety of the plant soot was evaluated through the test of 90-day subchronic toxicity, reproductive function, embryo developmental toxicity, and prenatal developmental toxicity for the first time. All the experiments were conducted with the purpose of confirming the non-toxicity and non-pathogenicity of the plant soot used as a novel feed additive and veterinary drug provide guidance on daily additive amount or clinical protocols and promote the safe application and effective promotion of plant soot in animal husbandry.

## Materials and Methods

### Chemicals

Plant soot (Lot#2-9243-2-01-11; purity 95%) was provided by China National Center for Veterinary Drug Safety Assessment (Beijing, China). 2,4,6-Trinitrobenzene (≥99% pure, CAS: 88-89-1) was obtained from Beijing Giant-Carrier Co., Ltd. (Beijing, China); 37–40% formalin and carboxyl methyl cellulose sodium (CMC-Na) were purchased from Tianjin Damao Chemical Reagent Factory Co., Ltd. (Tianjin, China). Potassium hydroxide was purchased from Shantou Xilong Chemical Plant Co., Ltd. (Shantou, China). Ethyl alcohol, xylene, paraffin, neutral gum, transparent agent, hematoxylin, eosin, chloral hydrate, citric acid, chromium potassium sulfate, glacial acetic acid, glycerin, and chloral hydrate were obtained from Sinopharm Chemical Reagent Co., Ltd. (Beijing, China). Alizarin red and sodium hydrochloride were purchased from Beijing Chemical Reagent Co., Ltd. (Beijing, China). Serum albumin (Alb), alanine aminotransferase (ALT), aspartate aminotransferase (AST), urea nitrogen (BUN), total cholesterol (TCH), creatinine (Cr), glucose (Glu), total protein (TP), and triglyceride (TG) detection kits were all obtained from Shanghai Kehua Bio-Engineering Co., Ltd. (Shanghai, China).

### Animals and Maintenance

Female and male Sprague Dawley (SD) rats were purchased from Beijing Vital River Laboratories. All rats were raised in the animal house of the Animal Veterinary Medicine Safety Evaluation Center of Ministry of Agriculture (Beijing) for 7 days to make an adjustment to the standard laboratory conditions including room temperature (20–26°C), relative humidity (50–65%), and artificial lighting (12-h light/dark cycle). All rats were supplied with sufficient rodent standard feed and water and kept free to eat and drink throughout the testing period. All facilities were avoided from being contaminated by exogenous factors, and kept clean during feeding and experiments. All rats were fasted overnight before the toxicity test but were not restricted to water. These animal experimental schemes have been ratified by the China Agricultural University Institutional Animal Care and Use Committee.

### Design of Subchronic Toxicity Test

A total of 80 rats aged 4–5 weeks (body weight, 70–90 g) were divided into four groups with 20 rats in each group (half male and female). The rat feed (purchased from Beijing Keao Xieli Feed Co., Ltd.) was added with plant soot of 0 mg/kg (negative control group), 2,000 mg/kg (low-dose), 10,000 mg/kg (medium-dose) and 50,000 mg/kg (high-dose) and was stirred in the feed mixer. Then the mixture was made into pellet feed used to be experimental diet of rats. After continuous oral administration for 90 days, the health status of the rats in the control and experimental groups was observed once a day, and the food consumption and body weight of all rats were recorded every 5 days. On the 90th day of the experiment, five male rats and five female rats were randomly selected from each group for the blood routine and biochemical examination, followed by weighing and dissecting. Hematological parameters were detected by Coulter-JT automatic blood routine detector, including hemoglobin (HGB), red blood cell (RBC), white blood cell (WBC), platelet (PLT), hematocrit (HCT), eosnophils (EOS), basophilic granulocyte (BAS), neutrophilic granulocyte (NEU), monocyte (MO), and lymphocyte (LYM). Biochemical parameters were examined by specific kits. Roch-Hitachi Modular automatic biochemical analyzer detected Alb, ALT, AST, BUN, TCH, Cr, Gl, TP, and TG. Next, some fresh organs were used for the calculation of organ coefficients and observation of pathological sections. Hematoxylin-eosin (HE)-stained rat liver, spleen, lung, heart, kidney and intestine were pathologically observed in all rats. The above experimental methods were all conducted according to the Ministry of Agriculture, PR China Notice No. 1247-8 (Guidelines for veterinary drug 30- and 90-day feeding tests) and modified based on relevant literatures.

### Design of Reproductive and Embryonic Toxicity Test

A total of 120 rats aged 7–8 weeks including 80 female rats (body weight, 220–250 g) and 40 male rats (body weight, 250–300 g) were prepared. The ratio of male to female in the same cage was 2:1. The female rats with sperm in the vaginal smear were selected as the newly “conceived” rats (not <12 conceived rats). The plant soot was configured into suspension by 2% CMC-Na solvent. Seven-day pregnant female rats were randomly divided into four groups, in which the experimental groups were administrated plant soot *via* intragastric gavage (IG) at 312.5 mg/kg (low-dose), 1,250 mg/kg (medium-dose), and 5,000 mg/kg bw (high-dose) suspensions (0.6 ml/10 g bw), respectively, and the negative control group received IG 2% CMC-Na solvent (0.6 ml/10 g bw). All pregnant rats were treated for 10 consecutive days, three times within a 24-h interval. As a last step, the rats were dissected on their 20th day of pregnancy, then the reproductive function indexes, embryonic development indexes, and fetal deformity indicator were detected and analyzed. The above experimental methods were all conducted according to the Ministry of Agriculture, PR China Notice No.1247-13(Guidelines for veterinary Drug Traditional Teratogenic tests in rats) and modified based on relevant literatures.

### Statistical Analysis

All values were presented as mean ± standard deviation (mean ± SD). Statistical analysis was conducted using the SPSS 20.0 by *t*-test. The data were compared with the same sex of control group. The significance level was considered at ^*^p < 0.05.

## Results

### Clinical Parameters

**Figure 2:**
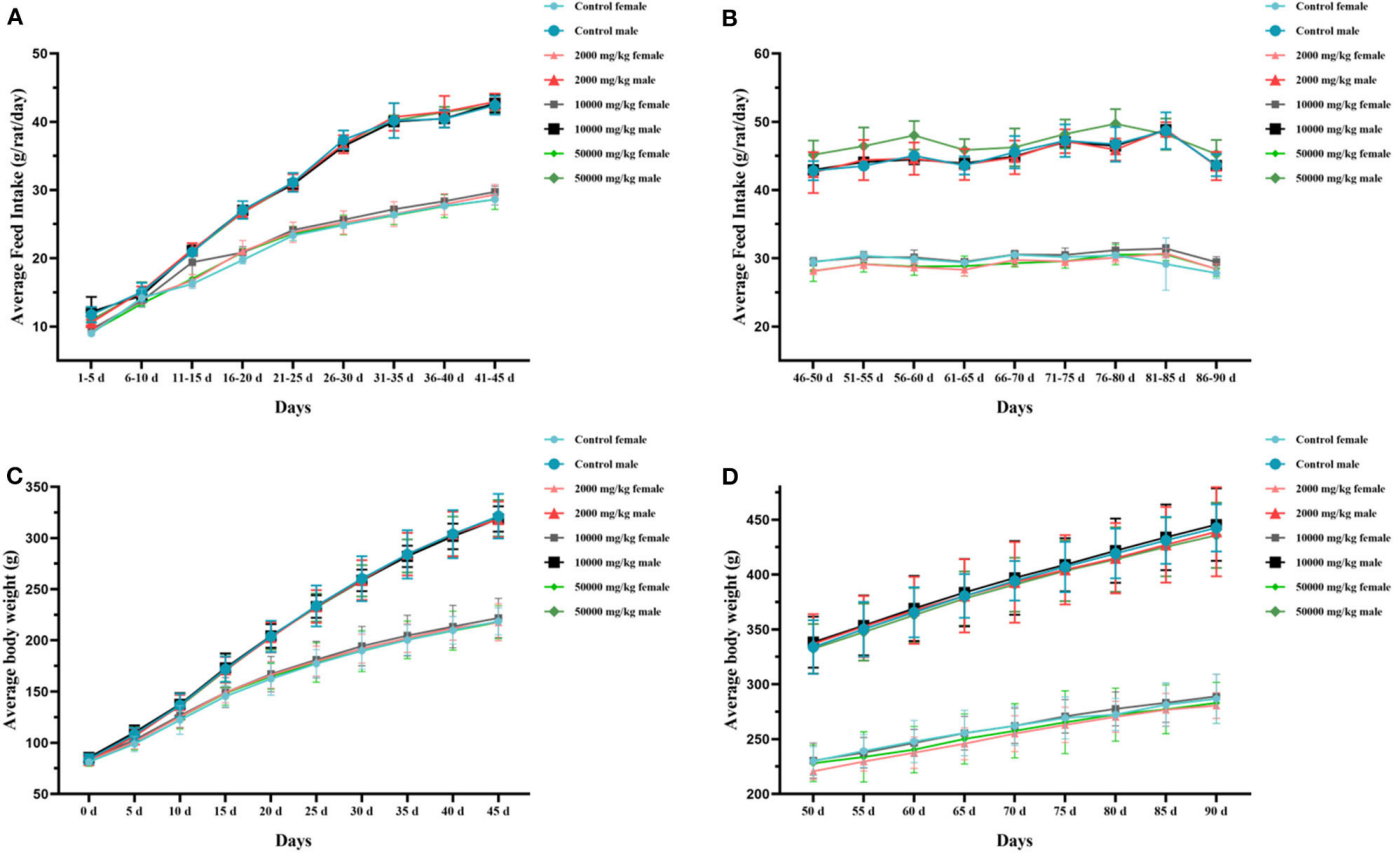
Effect of plant soot on average feed intake and average body weight of rats in the 90-day feeding study, as follows: **(A)** 1–45 days feed intake, **(B)** 46–90 days feed intake, **(C)** 1–45 days body weight, and **(D)** 50–90 days body weight. No significant difference was observed.

The rats in both the test groups and the control group survived to the end of the administration and recovery periods without moribund. No side-effect-related clinical signs or behavioral abnormalities were observed during the 90-day study. The hearing ability, pupillary reflex, hair luster, eye discharge, nasal discharge, and excrement were normal in all examined rats. The statistical changes in the feed intake and body weight of rats in 90 days are summarized in Figure 2, indicating that there was no significant difference between the treatment groups and the control group. Thus, it could be asserted that self-help feeding of pellet feed supplemented with 2,000–50,000 mg/kg bw plant soot had no significant effect on the growth or daily food consumption of rats within 90-day subchronic toxicity test.

### Hematology and Serum Biochemistry

Compared with the control group in hematology analysis (Table 2), the rats treated with plant soot showed no significant differences with respect to hematological parameters, such as the RBC, HGB, WBC, PLT, HCT, as well as the counts of EOS, BAS, NEU, MO, and YM. Data obtained from serum biochemical analyses are revealed in Table 3 indicating that the serum biochemistry levels of the test group rats including the Alb, ALT, AST, BUN, TCH, Cr, Glu, TP, and TG did not substantially differ from those in the control group. Therefore, no statistically significant changes in hematology and serum biochemistry parameters were brought from 90-day plant soot treatment.

**Table 2:** Effect of plant soot on hematological parameters of rats after feeding for 90 days.

**Groups**	**Blood parameters**																			
	**HGB (g/L)**		**RBC (M/mm**^****3****^**)**		**WBC (th/mm**^****3****^**)**		**PLT (th/mm**^****3****^**)**		**HCT (%)**		**EOS (G/L)**		**BAS (G/L)**		**NEU (10**^****9****^**/L)**		**MO (G/L)**		**LYM (G/L)**	
	**♀**	**♂**	**♀**	**♂**	**♀**	**♂**	**♀**	**♂**	**♀**	**♂**	**♀**	**♂**	**♀**	**♂**	**♀**	**♂**	**♀**	**♂**	**♀**	**♂**
50,000 mg/kg	144.81 ± 5.57	149.42 ± 9.53	8.92 ± 1.78	8.45 ± 0.55	8.72 ± 1.69	10.61 ± 1.19	391.55 ± 52.66	396.82 ± 55.05	45.67 ± 2.01	48.54 ± 3.81	0.03 ± 0.03	0.07 ± 0.05	0.12 ± 0.12	0.07 ± 0.05	3.53 ± 0.78	2.82 ± 1.05	0.25 ± 0.11	0.18 ± 0.04	4.22 ± 0.82	4.62 ± 1.35
10,000 mg/kg	145.92 ± 8.79	143.87 ± 9.68	8.49 ± 0.43	8.10 ± 0.74	8.52 ± 0.81	11.77 ± 1.29	371.14 ± 51.94	380.5 ± 56.76	46.45 ± 3.84	45.49 ± 3.91	0.06 ± 0.05	0.08 ± 0.06	0.08 ± 0.03	0.10 ± 0.08	3.59 ± 1.17	2.83 ± 1.19	0.24 ± 0.1	0.25 ± 0.11	4.05 ± 0.9	4.26 ± 0.9
2,000 mg/kg	148.93 ± 3.03	145.01 ± 9.05	8.09 ± 0.86	8.49 ± 0.71	8.51 ± 0.42	10.56 ± 1.49	397.51 ± 47.72	391.17 ± 48.22	46.37 ± 3.40	45.35 ± 2.19	0.11 ± 0.04	0.08 ± 0.04	0.05 ± 0.01	0.08 ± 0.05	4.26 ± 0.22	3.18 ± 0.88	0.24 ± 0.1	0.24 ± 0.12	4.18 ± 0.67	4.16 ± 1.19
Control	145.79 ± 8.31	148.21 ± 9.26	6.96 ± 0.88	8.51 ± 0.58	6.56 ± 1.19	9.93 ± 1.04	367.99 ± 68.11	380.62 ± 63.28	45.62 ± 3.01	47.67 ± 3.02	0.12 ± 0.07	0.08 ± 0.02	0.07 ± 0.05	0.08 ± 0.06	3.78 ± 0.69	3.45 ± 0.74	0.20 ± 0.15	0.19 ± 0.11	3.86 ± 1.84	4.32 ± 1.20

*The data were compared with the same sex of control group. Statistical analysis was conducted using the SPSS 20.0 by t-test*.

*HGB, hemoglobin; RBC, red blood cell; WBC, white blood cell; PLT, platelet; HCT, hematokrit; EOS, eosnophils; BAS, basophilic granulocyte; NEU, neutrophilic granulocyte; MO, monocyte; LY, lymphocyte. All values were presented as mean ± SD*.

* *p < 0.05, significance level*.

**Table 3:** Effects of plant soot on biochemical parameters of rats after feeding for 90 days.

**Groups**	**Biochemical parameters**																	
	**Alb (mmol/L)**		**ALT (U/L)**		**AST (U/L)**		**BUN (mmol/L)**		**TCH (mmol/L)**		**Cr (μmoL/L)**		**Glu (mmol/L)**		**TP (G/L)**		**TG (mmol/L)**	
	**♀**	**♂**	**♀**	**♂**	**♀**	**♂**	**♀**	**♂**	**♀**	**♂**	**♀**	**♂**	**♀**	**♂**	**♀**	**♂**	**♀**	**♂**
50,000 mg/kg	36.24 ± 0.94	37.69 ± 3.74	57.15 ± 8.64	54.87 ± 4.27	266.21 ± 28.9	218.56 ± 42.86	5.02 ± 0.71	4.53 ± 1.28	1.61 ± 0.30	1.74 ± 0.12	40.75 ± 8.59	50.52 ± 5.14	4.65 ± 0.92	4.55 ± 0.89	76.52 ± 3.66	75.22 ± 3.13	1.40 ± 0.07	1.33 ± 0.02
10,000 mg/kg	36.60 ± 2.50	34.82 ± 2.99	56.12 ± 5.11	52.5 ± 8.15	238.64 ± 28.16	215.71 ± 14.90	5.19 ± 0.78	5.01 ± 0.64	1.43 ± 0.17	1.52 ± 0.33	41.51 ± 6.79	47.81 ± 5.76	4.84 ± 0.93	4.68 ± 1.32	76.51 ± 4.10	73.44 ± 2.99	1.42 ± 0.07	1.35 ± 0.05
2,000 mg/kg	34.99 ± 2.74	36.27 ± 1.74	58.34 ± 6.49	53.88 ± 3.68	231.99 ± 40.17	243.42 ± 34.21	4.82 ± 1.29	4.86 ± 0.58	1.58 ± 0.24	1.52 ± 0.33	44.94 ± 5.49	45.72 ± 3.01	4.81 ± 0.64	4.9 ± 0.72	74.16 ± 2.40	74.80 ± 3.75	1.34 ± 0.04	1.36 ± 0.03
Control	35.99 ± 2.76	34.68 ± 1.83	52.34 ± 8.51	51.57 ± 5.05	251.65 ± 24.65	239.72 ± 26.02	4.36 ± 1.57	5.38 ± 0.69	1.85 ± 0.09	1.66 ± 0.20	48.14 ± 5.20	45.11 ± 3.44	6.01 ± 0.61	5.44 ± 0.89	74.99 ± 2.90	75.02 ± 4.50	1.38 ± 0.03	1.37 ± 0.10

*All values were presented as mean ± SD. The data were compared with the same sex of control group. Statistical analysis was analyzed using the SPSS 20.0 by t-test*.

*Alb, albumin; ALT, alanine aminotransferase; AST, aspartate aminotransferase; BUN, urea nitrogen; TCH, total of cholesterol; Cr, creatinine; Glu, glucose; TP, total protein; TG, triglyceride*.

* *p < 0.05, significance level*.

### Organ Coefficient and Histopathology

**Figure 3:**
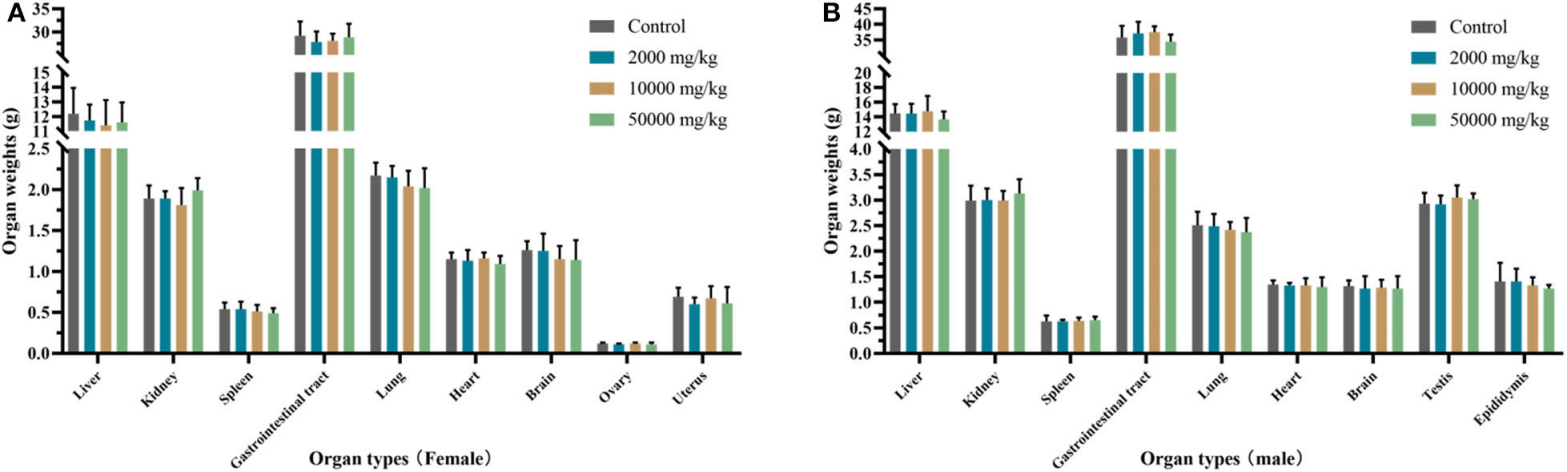
Effect of plant soot on organ weight of rats after feeding for 90 days. **(A)** Organ weights of female. **(B)** Organ weights of female. No significant difference was observed.

**Figure 4:**
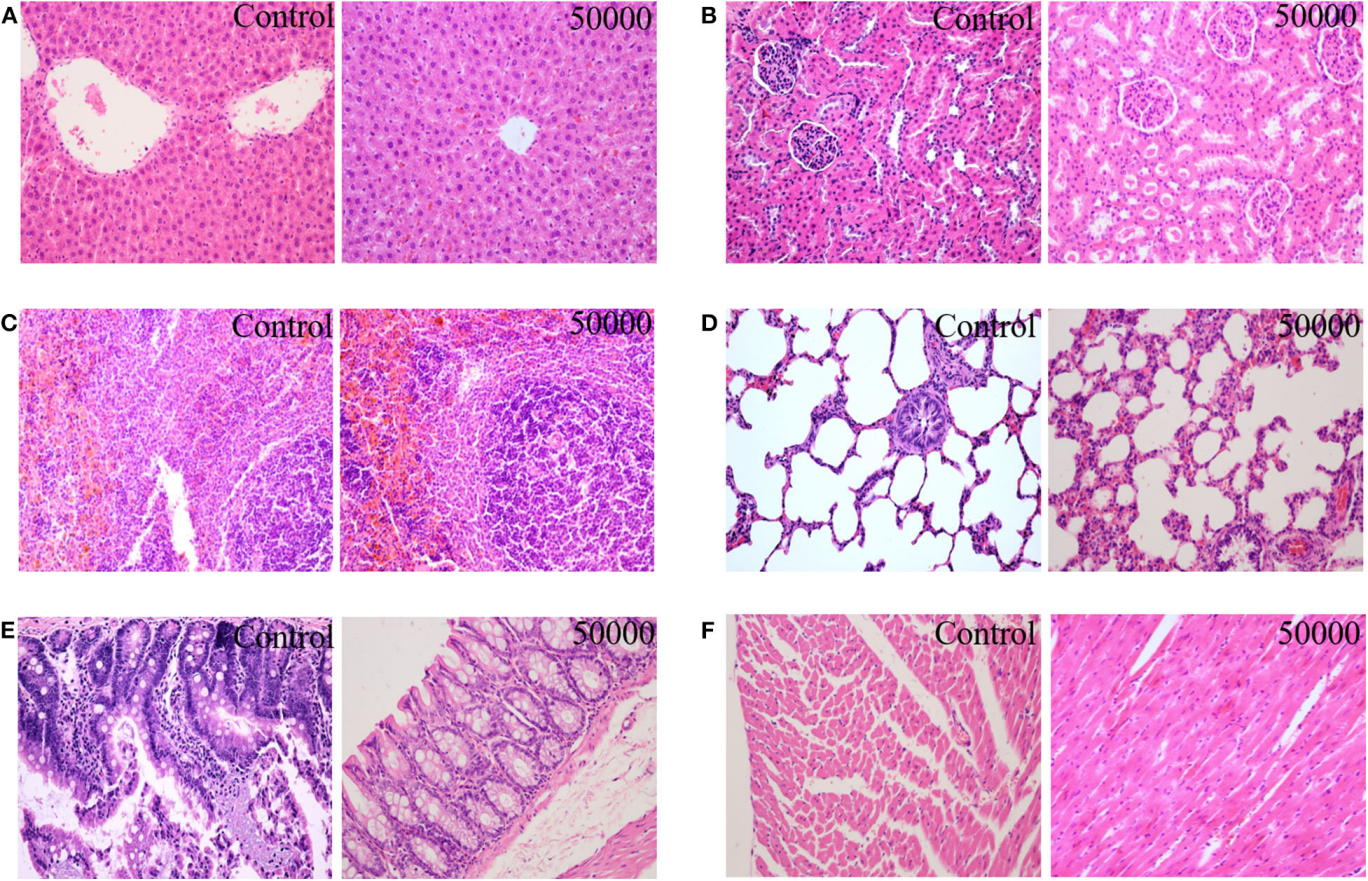
Effect of plant soot on the histopathological change in rats after feeding for 90 days (female and male were similar, only the control group and the high-dose group were included); **(A)** liver; **(B)** kidney; **(C)** spleen; **(D)** lung; **(E)** intestine; **(F)** heart.

The organ weights of heart, liver, kidney, spleen, lung, gastrointestinal tract, testis, and ovary in dissected rats were detected, showing that they were nearly equal in control, low-dose, medium-dose, and high-dose groups (Figure 3). More directly, there were no statistically significant alterations of organ coefficients (ratio of organ to body weight at day 90) in the treatment groups and control group (Table 4). Furthermore, HE-stained rat liver, spleen, lung, and other important tissue sections were pathologically observed in all rats, and no visible histopathologic differences were found even between the highest-dose groups and control group (Figure 4), meaning that 50,000 mg/kg bw plant soot added in the daily feed had no significant effect on the morphological information in various rat organs.

**Table 4:** Effect of plant soot on organ coefficient of rats after feeding for 90 days.

**Groups**	**Organ coefficient**																	
	**Liver**		**Kidney**		**Spleen**		**Gastrointestinal**		**Lung**		**Heart**		**Brain**		**Testis**	**Epididymis**	**Ovary**	**Uterus**
	**♀**	**♂**	**♀**	**♂**	**♀**	**♂**	**♀**	**♂**	**♀**	**♂**	**♀**	**♂**	**♀**	**♂**				
50,000 mg/kg	4.22 ± 0.67	3.14 ± 0.67	0.72 ± 0.07	0.72 ± 0.10	0.17 ± 0.02	0.15 ± 0.01	10.52 ± 1.80	7.93 ± 0.50	0.73 ± 0.08	0.54 ± 0.02	0.39 ± 0.05	0.30 ± 0.04	0.42 ± 0.1	0.30 ± 0.06	0.70 ± 0.04	0.30 ± 0.03	0.04 ± 0.01	0.44 ± 0.03
10,000 mg/kg	4.03 ± 0.50	3.37 ± 0.47	0.64 ± 0.09	0.68 ± 0.06	0.20 ± 0.02	0.15 ± 0.02	9.95 ± 0.61	8.57 ± 0.56	0.72 ± 0.09	0.55 ± 0.01	0.41 ± 0.04	0.30 ± 0.02	0.41 ± 0.09	0.30 ± 0.03	0.70 ± 0.09	0.31 ± 0.04	0.04 ± 0	0.44 ± 0.04
2,000 mg/kg	4.15 ± 0.46	3.31 ± 0.37	0.67 ± 0.05	0.69 ± 0.08	0.19 ± 0.06	0.16 ± 0.03	9.87 ± 1.17	8.50 ± 0.48	0.76 ± 0.06	0.57 ± 0.03	0.4 ± 0.05	0.30 ± 0.02	0.45 ± 0.07	0.30 ± 0.07	0.67 ± 0.05	0.33 ± 0.06	0.04 ± 0.01	0.46 ± 0.07
Control	4.52 ± 0.93	3.18 ± 0.32	0.69 ± 0.05	0.66 ± 0.08	0.21 ± 0.05	0.13 ± 0.02	10.78 ± 1.62	7.87 ± 0.92	0.80 ± 0.07	0.55 ± 0.08	0.42 ± 0.04	0.30 ± 0.03	0.47 ± 0.07	0.29 ± 0.04	0.65 ± 0.08	0.31 ± 0.07	0.04 ± 0.01	0.45 ± 0.03

*Organ coefficient = organ weight/body weight × 100%. All values were presented as mean ± SD. The data were compared with the same sex of control group. Statistical analysis was analyzed using the SPSS 20.0 by t-test*.

* *p < 0.05, significance level*.

### Reproduction and Embryonic Development

The differences in daily body increments of pregnant rats were found to be non-significant from day 0 of pregnancy till the end of the test in plant soot-treated groups compared with the control group. The details in Table 5 showed that average of net weight gains fluctuated slightly due to random errors but with no significant effects on judging the results. Table 6 demonstrated that parameters of reproductive effects, pointing out that the corpus luteum levels, ovary weight, and uterus weight in treated rats with 312.5–5,000 mg/kg plant soot roughly unchanged compared with the control. In addition, the non-interference of the plant soot on the rate of embryo mortality, survival, and absorption was also obtained in Table 7. Also, regarding the number which could represent the development of the embryo such as the placenta weight, fetal weight, fetal length, and fetal tail length, all showed no obvious responses to the plant soot treatment (Table 7).

**Table 5:** Effect of plant soot on daily weight gain in pregnant rats.

**Groups**	**Number of pregnancies**	**Pregnant rats gain weight**					
		**0–6 days**	**7–12 days**	**13–16 days**	**16–20 days**	**0–20 days**	**Net weight**
5,000 mg/kg	12	2.92 ± 0.46	3.76 ± 0.54	5.97 ± 0.77	8.27 ± 1.46	4.75 ± 0.41	1.41 ± 0.28
1,250 mg/kg	12	2.92 ± 1.46	4.49 ± 0.88	5.94 ± 1.75	7.19 ± 1.73	4.85 ± 0.40	1.18 ± 0.42
312.5 mg/kg	12	2.88 ± 1.11	3.92 ± 1.08	5.92 ± 1.18	6.40 ± 0.26	4.96 ± 0.26	1.34 ± 0.26
Control	12	2.81 ± 0.95	4.19 ± 1.40	6.65 ± 1.88	8.00 ± 1.54	5.03 ± 0.55	1.33 ± 0.30

*All values were presented as mean ± SD. The data were compared with the same sex of control group. Statistical analysis was analyzed using the SPSS 20.0 by t-test*.

* *p < 0.05, significance level*.

**Table 6:** Effect of plant soot on reproductive function of rats.

**Groups**	**Number of pregnancies**	**Ovary weight (g)**	**Number of corpus luteum**	**Uterus weight (g)**
5,000 mg/kg	12	0.13 ± 0.02	13.50 ± 2.11	5.98 ± 0.43
1,250 mg/kg	12	0.12 ± 0.02	13.08 ± 1.08	5.96 ± 0.65
312.5 mg/kg	12	0.12 ± 0.02	14.50 ± 1.38	5.98 ± 0.54
Control	12	0.13 ± 0.02	13.67 ± 1.37	5.60 ± 0.61

*All values were presented as mean ± SD. The data were compared with the same sex of control group. Statistical analysis was analyzed using the SPSS 20.0 by t-test*.

* *p < 0.05, significance level*.

**Table 7:** Effect of plant soot on survival rate of embryo and embryonic development of rats.

**Groups**	**Number of pregnancies**	**Number of implants**	**Number of live born**	**Embryo absorption (%)^a^**	**Embryo mortality (%)^b^**	**Embryo survival (%)^c^**	**Placenta weight (g)**	**Fetal weight (g)**	**Fetal length (cm)**	**Fetal tail length (cm)**
5,000 mg/kg	12	138	137	0.72 (1/138)	0 (0/138)	99.28 (137/138)	0.48 ± 0.02	3.62 ± 0.10	3.65 ± 0.12	1.22 ± 0.04
1,250 mg/kg	12	140	139	0.71 (1/140)	0 (0/140)	99.29 (139/140)	0.49 ± 0.02	3.65 ± 0.03	3.70 ± 0.03	1.23 ± 0.02
312.5 mg/kg	12	131	131	0 (0/131)	0 (0/131)	100 (131/131)	0.51 ± 0.01	3.69 ± 0.07	3.70 ± 0.03	1.24 ± 0.02
Control	12	131	130	0.76 (1/131)	0 (0/131)	99.24 (130/131)	0.49 ± 0.05	3.67 ± 0.10	3.71 ± 0.01	1.23 ± 0.01

*All values were presented as mean ± SD. The data were compared with the same sex of control group. Statistical analysis was analyzed using the SPSS 20.0 by t-test*.

* *p < 0.05, significance level*.

a *Number of absorption/number of implants*.

b *Number of stillbirth/number of implants*.

c *Number of live born/number of implants*.

### Teratological Detection

**Figure 5:**
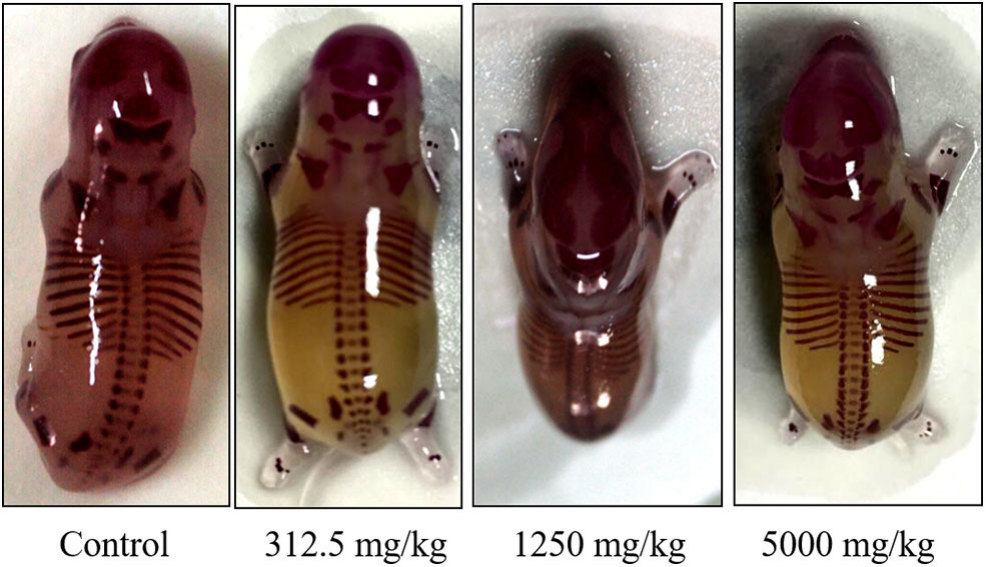
Effect of plant soot on skeletal malformations in rats.

There was no significant difference in the incidence of fetal malformation and maternal malformation between the test groups and the control (Table 8), in which the malformation rate of appearance and viscera were both 0%. Obtained fetal rats from treated and control groups were inspected for skeletal malformations and shown in Figure 5. Within the normal historical range of our laboratory, about 5% of rat embryos have skeletal deformities. The embryo shape of experimental rats in the highest-dose group was normal with the growth of multiregional skeleton in good situation, giving the evidences that plant soot made no obvious teratogenic impact on the appearance, internal organs, and skeleton of fetal rats.

**Table 8:** Teratogenic effect of plant soot on rats.

**Groups**	**Number of pregnancies**	**Malformation rate of appearance**			**Malformation rate of skeleton**			**Malformation rate of internal organs**		
		**Number of fetal rats**	**Teratogenesis rate of fetal rats (%)^a^**	**Maternal malformation rate (%)^b^**	**Number of fetal rats**	**Teratogenesis rate of fetal rats (%)^a^**	**Maternal malformation rate (%)^b^**	**Number of fetal rats**	**Teratogenesis rate of fetal rats (%)^a^**	**Maternal malformation rate (%)^b^**
5,000 mg/kg	12	137	0	0	75	5.33 (4/75)	16.67 (2/12)	62	0	0
1,250 mg/kg	12	139	0	0	80	2.50 (2/80)	0 (0/12)	59	0	0
312.5 mg/kg	12	131	0	0	77	6.49 (5/77)	16.67 (2/12)	54	0	0
Control	12	130	0	0	81	4.94 (4/81)	25.00 (3/12)	49	0	0

a *Number of deformities/number of fetal rats*.

b *Number of deformities/number of pregnancies*.

## Discussion

It has been known that overused antibiotics in animal feed could lead to the development of drug-resistant bacteria and drug residues as threats to human health, so they have been phased out as growth promoters in many countries (12–14). On the other hand, there is increasing value in finding natural and harmless feed additives to strengthen the immune system and fight against various diseases, thus new antibiotic substitution strategies and alternative feed additives such as microecological preparations and traditional herbal medicine are currently increasing in popularity (15–17). It reported that the dietary supplementation with propionibacteria promoted mucus secretion characterized by rising goblet cells counts, neutral mucin production, and the longer villus-crypt units. The improvement of intestinal microbial activity of poultry was also the evidence of probiotic potential of the Propionibacterium (18). Furthermore, it was observed that Enterobacteria-like *Salmonella*/*Shigella* in goats were reduced by probiotics, to be replaced by decreased Bifidobacterium, lactic acid bacteria, and fecal mutagen, proving that oral administration of probiotics had an effective protective effect on the digestive system of goats (19). Since traditional Chinese medicine was easier to obtain and store compared with microecological preparations (20), the effects of herbal feed additives has been evaluated in the previous literatures. The growth performance and immunity of pigs were improved by preparations of herbal medicine compound. Compared with the antibiotic groups, the pigs in the experimental groups displayed higher average intake, higher HDL level, higher peripheral blood CD3+CD8+ T cell ratio, and lower LDL level (21). In addition, the effect of single herb on aquaculture has also been demonstrated. For example, the resistance of common carp to potential pathogens such as *Aeromonas hydrophila* was significantly improved during the consumption of *Astragalus* root extracts (22). The intestinal flora of crucian carps and the microbial community in the water were balanced by the Jade screen powder in feed, accompanied by effective improvement of the lactic acid bacteria, *Lactococcus, Bacillus*, and other beneficial bacteria, as well as the decline of the probability of *Aeromonas* and *Acinetobacter* infection (23). All the evidences have pointed to new feed additives, especially herbal medicine products, as one of the most popular and effective alternatives to antibiotics.

Plant soot, one of the herbal medicine products, has just been approved as a novel natural feed additive to adsorb intestinal mycotoxin, inhibit bacterial reproduction, and improve the digestive function of animals without data published concerning its dosage and safety (3). Since the plant soot originated from the carbonized and activated products of the Chinese fir bits, pine bits, or bamboo bits, it has the advantage of easy access and low cost compared with other new feed additives. It has been reported that the intestinal flora of animals was balanced, the symptoms of diarrhea were reduced, and the growth of animals was promoted through either using plant soot feed as additives in animal husbandry or applying plant soot to drug combination in veterinary clinic (8). Most importantly, this natural feed additive has made full use of wood waste after carbonization. For instance, wide variety of sawdust were collected in wood processing sites, then they were carbonized and activated into plant soot, and the feed additives was produced through the complex process of sieving, cleaning, and inspection, becoming the industrialization of plant soot. The product subsequently exerted beneficial effects in animal gastrointestinal tract, mainly about adsorbing toxins hiding in intestinal contents, balancing microbiology, and improving digestion. Eventually, the plant soot traveled into nature along with the excrement, preparing for the next cycle. It has been well-known that all kinds of black carbon from combustion have worsened global pollution and posed a lot of challenges for humans and animals (24). However, some previous researches have shown that cleverly exploiting the natural products from carbonization could also reduce ecological risk and health crisis. The carbon soot recycled from diesel particulate filters has been proved to potentially adsorb copper, cadmium, and other heavy metals from wastewater (11). There is also the concept of the natural adsorbent, which has been applied to adsorb aflatoxin B1 in *in vivo* and *in vitro* (25). Whereas, the only natural product that has combined sawdust carbonization with adsorption and was used for animal growth and disease treatment is currently plant soot. It needs to be clear that the disservice of plant carbon to humans has changed from absolute to relative, for the reason that the toxicity test results and other safety evaluation result of the novel feed additive, plant soot, was positive going. Therefore, the results of this experiment could be applied to macroenvironmental toxicology.

Although plant soot has been known as a natural product, with no side effects or adverse reactions reported, detailed toxicological identification and safety evaluation are still required before mass marketing. Up to now, there was no literature that reported on evaluation and analysis about the toxicity of plant soot, and the only existing analyses are mainly aimed at the medical value of plant soot, without the evaluation and analysis of toxicity either *in vivo* or *in vitro*. Herein, a repeated-dose (90-day) oral toxicity and a prenatal developmental study *in vivo* were conducted to test the safety of plant soot for use as rat feed ingredient. In subchronic test, the life indication and behavior of all rats were stable during the 90 days; no signs of moribund and toxicity were observed for any rats after the experimental period. The rats in the treatment and control groups all exhibited daily body weight increments, while there were no significant differences between these two groups with respect to food consumption and body weight. Although the blood system, liver function, and kidney function were known to be all highly sensitive to exogenous substance, there was no apparent difference in hematology and serum biochemistry between rats of administration groups and the control. All cells and indexes associated with infections and diseases remained within the normal range. Therefore, plant soot did not cause substantial threat to the rat immune systems. Another index, organ coefficients of heart, liver, kidney, spleen, lung, gastrointestinal tract, testis, and ovary in dissected rats were detected and showed no statistically significant variations in the treated rats and the control. Besides, there were no alterations among groups in the aspect of color, size, and other microscopic appearance characteristics in rat liver, spleen, lung, and other important tissue sections. It suggested that 50,000 mg/kg plant soot added in the daily feed was the maximum NOEL of subchronic toxicity test. It has been widely acknowledged that embryos or newborns were both sensitive to toxic substances, so the index of reproductive function, embryo development, and teratogenicity indexes could be used as a more direct evidence to clarify the safety of plant soot. In prenatal developmental toxicity study, the differences in daily body increments between plant soot-treated rats and control rats were found to be non-significant from day 0 of pregnancy to the end of the experiment. The non-interference of the plant soot on parameters of reproductive indication of corpus luteum levels, ovary weight, and uterus weight in rats was confirmed. In addition, the rate of embryo mortality, survival, and absorption in the plant soot-treated rats was roughly unchanged compared with the control. The number that represented the development of embryo including placenta weight, fetal weight, fetal length, and fetal tail length also exhibited no obvious responses to the plant soot. Furthermore, there was no significant difference in the incidence of fetal malformation and maternal malformation between the test groups and the control group in terms of appearance, internal organs, and skeleton. It could be illustrated that pregnant rats and fetus were exposed to the plant soot without any toxicity, and the maximum NOEL of embryo teratogenicity test could be set to 5,000 mg/kg. In addition, more comprehensive toxicity test needs to be conducted and combined with other animal models in the future to fully prove the non-toxic and harmless characteristics of this novel natural feed additive.

## Conclusion

**Figure 6:**
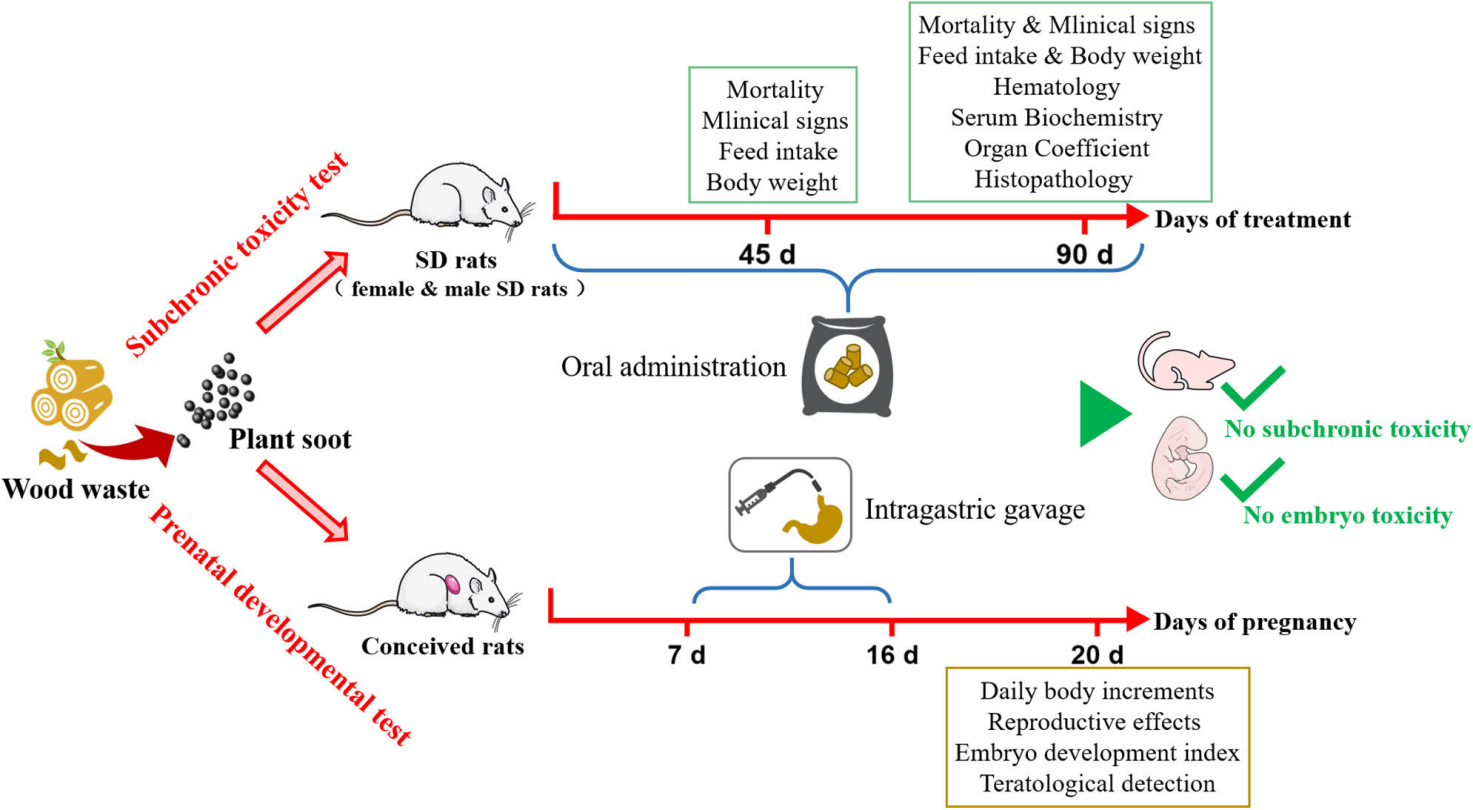
Safety assessment for subchronic toxicity or prenatal developmental toxicity of plant soot.

In summary, detailed safety assessment results of plant soot were presented in this study, and no significant subchronic toxicity or teratogenic toxicity was found (Figure 6). The maximum NOEL of supplemental dosage in feed for subchronic toxicity test could be set to 50,000 mg/kg, and the intragastric maximum NOEL of teratogenic test could be set to 5,000 mg/kg. This conclusion has suggested the dosing regimen of plant soot in the daily growth or clinical treatment of animals and will provide the data background for further pre-clinical safety evaluation and clinical trial design of plant soot. Treated rats all had normal physical and behavioral indicators after exposing to oral plant soot for 90 days, and the plant soot treatment of 10 consecutive days did not hurt any of the pregnant and fetal rats, showing that this novel kind of natural feed additive was relatively safe for animals. In the future, more toxicological studies will be appended, and the potential to become a standard or model for non-toxic feed additives will be further proved upon plant soot. Of course, all additions and usage should be treated with caution until the toxicity and risks of plant soot are fully assessed. Therefore, further toxicity and pharmacokinetic studies will be necessary to confirm the safety of plant soot in order to benefit the development of “green animal husbandry.”

## Data Availability Statement

The original contributions generated in the study are included in the article/supplementary materials, further inquiries can be directed to the corresponding author/s.

## Ethics Statement

The animal study was reviewed and approved by China Agricultural University Institutional Animal Care and Use Committee.

## Author Contributions

XP and DL contributed to the design of the experiments and wrote the manuscript. JL and LK contributed to the animal experiments and data analysis. XX and ST reviewed the manuscript. All authors have read and approved the final manuscript.

## Conflict of Interest

JL and LK were employed by the company of Fujian Shunchang Tanwawa Biological Technology Co., LTD, and Fujian Plant Soot Biotechnology Co., LTD. The remaining authors declare that the research was conducted in the absence of any commercial or financial relationships that could be construed as a potential conflict of interest.
